# Impact of diabetes mellitus on short-term prognosis, length of stay, and costs in patients with acute kidney injury: A nationwide survey in China

**DOI:** 10.1371/journal.pone.0250934

**Published:** 2021-05-03

**Authors:** Lishan Tan, Li Chen, Yan Jia, Lingyan Li, Jinwei Wang, Xiaoyan Huang, Qiong Luo, Li Yang, Zuying Xiong, on behalf of ISN AKF 0 by 25 China Consortium

**Affiliations:** 1 Department of Nephrology, Peking University Shenzhen Hospital, Shenzhen Peking University–The Hong Kong University of Science and Technology Medical Center, Shenzhen, Guangdong, China; 2 Renal Division, Department of Medicine, Key Laboratory of Renal Disease, Ministry of Health of China, Peking University First Hospital, Peking University Institute of Nephrology, Beijing, China; Kaohsiung Medical University Hospital, TAIWAN

## Abstract

**Background:**

International data suggest that people with diabetes mellitus (DM) are at increased risk for worse acute kidney injury (AKI) outcomes; however, the data in China are limited. Therefore, this study aimed to describe the association of DM with short-term prognosis, length of stay, and expenditure in patients with AKI.

**Methods:**

This study was based on the 2013 nationwide survey in China. According to the 2012 Kidney Disease: Improving Global Outcomes (KDIGO) and expanded criteria of AKI, 7604 patients with AKI were identified, and 1404 and 6200 patients were with and without DM, respectively. Clinical characteristics, outcomes, length of stay, and costs of these patients were compared. Multivariate regression analyses were conducted to evaluate the association of DM with mortality, failed renal recovery, length of stay, and costs.

**Results:**

Patients with AKI and DM were older, had higher male preponderance (61.9%), presented with more comorbidities, and had higher serum creatinine levels compared with those without DM. An apparent increase in all-cause in-hospital mortality, length of stay, and costs was found in patients with DM. DM was not independently associated with failed renal recovery (adjusted OR (95%CI): 1.08 (0.94–1.25)) and in-hospital mortality (adjusted OR (95%): 1.16 (0.95–1.41)) in multivariate models. However, the diabetic status was positively associated with the length of stay (*β* = 0.06, *p*<0.05) and hospital expenditure (*β* = 0.10, *p*<0.01) in hospital after adjusting for possible confounders.

**Conclusion:**

In hospitalized AKI patients, DM (*vs*. no DM) is independently associated with longer length of stay and greater costs, but is not associated with an increased risk for failed renal recovery and in-hospital mortality.

## Introduction

Acute kidney injury (AKI) occurs in approximately 10–15%, and the mortality is up to 3.1%–28.0% in hospitalized patients, especially in patients with AKI requiring dialysis treatment [1, 2]. The International Society of Nephrology launched the “0by25” global target to improve the diagnosis and treatment of AKI. According to this, no patient should die due to untreated acute kidney failure by 2025 [3]. As part of this initiative, a nationwide survey on AKI in adults was conducted in China to estimate the burden of AKI. Many previous studies established the association between AKI and mortality in patients with a pre-existing chronic condition [4–6]. Thus, finding determinants and remedies and subsequently improving the prognosis patients of AKI are imperative.

Diabetes mellitus (DM) has become a common disease in adults due to lifestyle changes and increased prevalence of obesity. Many studies demonstrated an increase in the prevalence of DM from 0.9% in 1980 to 12.8% in 2017 in China [7, 8]. However, the Risk Evaluation of cAncers in Chinese diabeTic Individuals: a IONgitudinal (REACTION) study found that only 34.7% of patients with DM receiving treatment had their conditions under control [9]. Several reasons might explain the poor control of DM in China, as follows: (a) the low treatment rate of DM-related complications [10]; (b) few patients followed the diet and exercise recommendations; and (c) some patients did not comply with the treatment recommendations from doctors or nurses [11]. Poor control of DM can directly cause a high risk of microvascular and macrovascular complications and lead to poor prognosis of patients [12].

In addition, DM is one of the major risk factors for the development of AKI [13–15]. Reportedly, 14.5% of patients with DM developed AKI during hospitalization in East Ethiopia [16], while the corresponding incidence in China was 9.4% [17]. Age-standardized rates of AKI in hospitalized patients increased by 139% among adults diagnosed with DM from 2000 to 2014 in the United States [18]. Previous studies demonstrated a higher AKI morbidity and mortality risk among patients suffering from DM [19], which was in accordance with the findings on diabetic veterans in the United States [20]. However, a recent study from the United Kingdom demonstrated that patients with DM and severe AKI were less likely to be followed up and had the same acute mortality as patients without DM [21]. Therefore, the influence of DM on patients with AKI is still ambiguous and requires in-depth research. In clinical practice, DM easily causes several complications, which not only affect the quality of life, but also cause tremendous health-care expenditures [22, 23]. In 2015, an estimated individual and global health expenditure due to diabetes was $825 billion [24]. Direct medical costs for patients with DM reached $9.1 billion in China [25]. In the United States, patients with AKI had increased hospitalization costs of $7933 and a prolonged length of stay of 3.2 days compared with hospitalized patients without AKI [26]. Nevertheless, studies regarding the prognosis and economic burden of hospitalized patients with AKI and DM are relatively rare in China.

The present study was performed to evaluate the impact of DM on outcomes and the economic burden of hospitalized patients with DM in China. Moreover, the study compared the clinical characteristics and short-term outcomes between patients with AKI with and without DM.

## Materials and methods

### Study population and design

This study was derived from the International Society of Nephrology (ISN) Acute Kidney Failure 0by25 China Consortiums, a nationwide survey on adults AKI in China [27]. The features of patients with AKI and ways to recognize and treat AKI in the clinical practice in China were described in the study. Doctors retrospectively screened patients’ records and discriminated AKI on the basis of changes in serum creatinine levels using the Laboratory Information System in each hospital. The national survey was designed and the nephrologists and renal fellows were trained to achieve the standard work process and thus collect reliable data of patients. Therefore, the data for this survey was all based on the hospital records of patients. The study was approved by the Ethics Committees of Peking University First Hospital (2014[729]) and conducted from May, 2014. Because this clinical study is a retrospective study to collect medical record of patients, so we did not obtain written consent before the start of the study. Additionally, the Clinical Research Ethic Committee waived the requirement for informed consent of this study in the ratification. The project included 44 study hospitals and originated from 22 provinces, municipalities, and autonomous regions and 4 geographical regions (north, northwest, southeast, and southwest), encompassing 82% of the country’s population in 2013. From each region, an academic hospital or a local hospital was randomly enrolled, and 2,223,230 patients were admitted to these 44 hospitals.

### Inclusion and exclusion criteria

**Fig 1:**
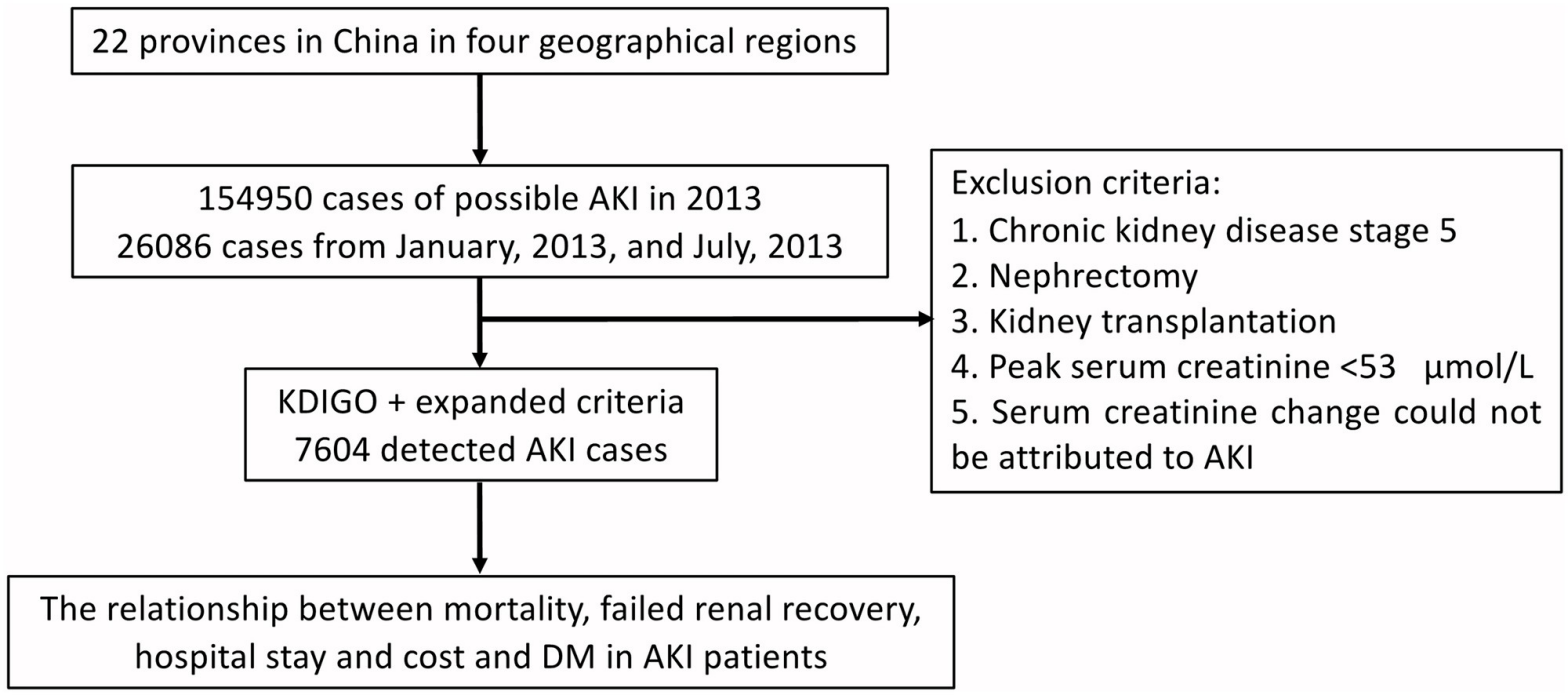
Study profile. AKI, acute kidney injury; DM, Diabetes mellitus.

Given the labor intensity of extracting AKI hospitalized information, we were only able to capture two months data in 2014. Some evidence has shown a seasonal effect of AKI [28], and thus in an attempt to approximate the average AKI across the year, we chose January (winter) and July (summer) months to examine effects between diabetes history and short clinical outcome in hospitalized AKI patients. Hence, only patients diagnosed with putative AKI in this survey between January and July 2013 were included. The AKI definition was mainly based on the following two criteria: (1) the 2012 Kidney Disease: Improving Global Outcomes (KDIGO) AKI definition (an increase in Scr level by 26.52 μmol/L or by 50% within 7 days, excluding urine output criteria) and (2) the AKI expanded criteria (an increase or decrease in serum creatinine level by 50% during hospitalization). The exclusion criteria were as follows: (1) chronic kidney disease stage 5, (2) nephrectomy, (3) kidney transplantation, (4) peak serum creatinine level<53 μmol/L, and (5) serum creatinine change could not be attributed to AKI. Finally, 7604 patients with AKI were enrolled in the following analysis; the study profile is presented in Fig 1. The Strengthening the Reporting of Observational Studies in Epidemiology (STROBE) statement is provided in (S1 Checklist) [29].

### Data collection

Data on the characteristics of patients with AKI cases were collected, including sociodemographic status, comorbidities [cardiovascular disease (CVD), hypertension (HBP), chronic kidney disease (CKD), and cerebrovascular disease (CEVD)], AKI classification, impairment factors (infection, crystalluria/cylindruria, operation, and so forth), nephrotoxic mediations (antibiotics, diuretics, and nonsteroidal anti-inflammatory drugs), peak serum creatinine level, serum creatinine level at discharge, peak AKI staging (1–3), critical condition (multiple-organ dysfunction, sepsis, terminal malignancy, shock, acute respiratory distress syndrome, mechanical ventilation, and thrombotic microangiopathy), renal replacement therapy (RRT) and RRT indication, and AKI recognition type (timely recognition, delayed recognition, and nonrecognition). The short-term prognosis (renal recovery, mortality, and treatment withdrawal), intensive care unit (ICU) and length of stay, and hospitalization costs of patients were compared between patients those with AKI with and without DM. The authors had access to information that could identify individual participants during or after data collection.

### Definitions of associated variables

DM was identified based on the following criteria: (1) data on the pre-existing diabetic condition, which were collected by doctors in the wards; (2) newly discovered DM by physicians during hospitalization, which was based on the diabetes-related symptom (polydipsia, polyuria, and more food and weight loss) and results of laboratory tests [fasting blood-glucose >7.0 mmol/L or 2-h plasma glucose or random plasma glucose >11.1 mmol/L or glycated hemoglobin A1c >48 mmol/mol (6.5%)], excluding some acute settings (acute infection or trauma, and so forth). However, no differentiate could be made between type 1 and type 2 DM. Briefly, patients with increased serum creatinine levels at admission or within 2 days after hospitalization, combined with causal factors that nephrologists determined those to be present before admission, were identified as patients with community-acquired AKI (CA-AKI). Others patients were recognized as those with hospital-acquired AKI (HA-AKI). Different categories of AKI were defined as follows: (1) prerenal, defined as decreased kidney perfusion (hypovolemia, decreased cardiac output/cardiorenal syndrome, peripheral vasodilatation, imbalance of renal vasoconstriction and vasodilatation, mechanical obstruction of renal artery); (2) intrarenal, defined as intrinsic kidney disease; and (3) postrenal, defined as urinary tract obstruction.

The peak AKI stage (1–3) was defined as the highest AKI stage reached by a patient during the whole in-hospital stay [30]. The diagnostic standards of the disease severity of AKI were shown in the S3 Table. AKI recognition was classified into three categories: timely recognition (AKI was diagnosed by the physicians in charge within 3 days and before the injury progressed to severer stages); delayed recognition (AKI was not diagnosed within 3 days); and nonrecognition (AKI was not recognized during the whole hospitalization). Next, the renal recovery was identified at discharge as follows: full recovery, serum creatinine level reduced to below threshold or to the baseline; partial recovery, serum creatinine decreased by ≥25% from the highest concentration but higher than the threshold or baseline; and failed recovery, patients dependent on dialysis or serum creatinine decreased < 25% from the peak level or death. Treatment withdrawal was confirmed as severely ill patients with AKI refusing further treatment due to personal or economic reasons. The primary outcomes were short-term prognosis, including all-cause in-hospital mortality and renal recovery. The secondary outcomes were length of stay and hospital costs. The hospital cost was identified with a total expenditure of one continuous turnover in an admission per patient. The main 10 items in the hospital cost were as follows: comprehensive medical services (medical service fee, treatment operation fee, and nursing fee), diagnostics (pathological diagnosis fee, laboratory diagnosis fee, and imaging diagnosis fee), treatment (non-surgical and surgical fee), rehabilitation, traditional Chinese medicine treatment, Western medicine, Chinese medicine, blood and blood products, consumables, and others.

### Statistical analysis

Continuous variables were described as means ± standard deviation (SD) or median (interquartile range, IQR), as appropriate. Categorical variables were reported as frequencies (proportions). The clinical characteristics, short-term prognosis, and length of stay of patients with AKI with and without DM were evaluated. Normally distributed data were analyzed using the Student *t*-test. The Wilcoxon rank-sum test was performed to compare the difference in skewed data. The groups were compared using the chi-square or Fisher’s exact test for categorical variables.

Before analyzing the association between DM history and length of stay and costs, the ultimate creatinine, length of stay, and hospital costs were log-transformed because of skewed distribution. Next, univariable and multivariable logistic regression models were used to further observe the odds of all-cause in-hospital mortality and failed renal recovery with DM history in patients with AKI. The relative covariates in multivariable logistic and linear models included age, sex (male or female), region (north, southeast, northwest, and southwest), CVD (yes or no), HBP (yes or no), CKD (yes or no), CEVD (yes or no), and tumor (yes or no). Patients with missing information on the dependent variables were excluded in the logistic and linear regression analyses.

Data were input and managed using Epidata software (version 3.1, Epidata Association, Odense, Denmark). A two-tailed *P* value <0.05 was considered statistically significant. All analyses were performed using SAS software (version 9.4, SAS Institute, Cary, NC, USA).

## Results

### Baseline characteristics in the DM and non-DM groups

A total of 7604 patients with AKI were enrolled in this study. The baseline characteristics of patients with AKI with and without DM are shown in Table 1. The majority of the patients with DM (74.7%) were aged ≥60 years and were older than patients without DM (68 ± 14 *vs*. 60 ± 18 years, *P* <0.001). Both groups comprised largely of men (>60%). Comorbidities, including CVD, HBP, CKD, were nearly two-folds in patients with DM compared to those without DM (*P* <0.001). Strikingly, prerenal AKI accounted for half of the cases in the two groups. A higher proportion of patients with DM developed intrarenal AKI compared with those without DM (30.8% *vs*. 26.9%). Among nephrotoxic drugs, nearly half of the patients with diabetes (46.8%) were more likely to use diuretics compared with those without diabetes (39.0%). Patients with DM presented higher levels of serum creatinine at both peak and discharge compared with those without DM [171.0 (122.1–266.5) μmol/L *vs*. 155.3 (113.0–253.0) μmol/L, 104.2 (73.2–161.9) μmol/L *vs*. 93.9 (67.0–143.0) μmol/L, *P* <0.001]. Few patients with DM were critical compared with those without DM (40.6% *vs*. 44.9%, *P* = 0.004). Additionally, no differences were detected in peak AKI staging and AKI recognition type between the two groups.

**Table 1:** Characteristics, short-term prognosis, length of stay, and costs of patients with AKI with and without DM.

Characteristics	Total (*n* = 7604)	Patients with DM (*n* = 1404)	Patients without DM (*n* = 6200)	*P*-value
Age (years)	62 ± 17	68 ± 14	60 ± 18	<0.001
Age group				
18–39	876 (11.5)	36 (2.6)	840 (13.6)	<0.001
40–59	2341 (30.8)	319 (22.7)	2022 (32.6)	
60–79	3120 (41.0)	748 (53.3)	2372 (38.3)	
≥80	1267 (16.7)	301 (21.4)	966 (15.6)	
Sex				
Male	4955 (65.2)	869 (61.9)	4086 (65.9)	0.004
Female	2649 (34.8)	535 (38.1)	2114 (34.1)	
Region				
North	2097 (27.6)	410 (29.2)	1687 (27.2)	0.01
Southeast	3406 (44.8)	580 (41.3)	2826 (45.6)	
Northwest	851 (11.2)	153 (10.9)	698 (11.3)	
Southwest	1250 (16.4)	261 (18.6)	989 (16.0)	
Source				
HA-AKI	3468 (45.6)	670 (47.7)	2798 (45.1)	0.078
CA-AKI	4136 (54.4)	734 (52.3)	3402 (54.9)	
Comorbidity				
Any comorbidity	4723 (62.1)	1159 (82.6)	3564 (57.5)	<0.001
CVD	2666 (35.1)	727 (51.8)	1939 (31.3)	<0.001
HBP	3190 (42.0)	952 (67.8)	2238 (36.1)	<0.001
CKD	1847 (24.3)	507 (36.1)	1340 (21.6)	<0.001
CEVD^a^	1137 (15.0)	334 (23.8)	1203 (13.0)	<0.001
AKI classification				
Pre-renal	3936 (51.8)	722 (51.4)	3214 (51.8)	0.783
Intra-renal	2100 (27.6)	432 (30.8)	1668 (26.9)	0.003
Post-renal	670 (8.8)	77 (5.5)	593 (9.6)	<0.001
Unclassified	898 (11.8)	173 (12.3)	725 (11.7)	0.51
Impairment factors				
Infection	4139 (54.4)	767 (54.6)	3372 (54.4)	0.869
Crystalluria/Cylindruria	55 (0.7)	10 (0.7)	45 (0.7)	0.949
Operation	1476 (19.4)	245 (17.5)	1231 (19.9)	0.04
Drugs				
Antibiotics	3499 (46.0)	656 (46.7)	2843 (45.9)	0.555
Diuretics	3072 (40.4)	657 (46.8)	2415 (39.0)	<0.001
NSAIDs	901 (11.9)	172 (12.3)	729 (11.8)	0.606
Traditional Chinese medicine	129 (1.7)	24 (1.7)	105 (1.7)	0.967
Contrast agents	681 (9.0)	135 (9.6)	546 (8.8)	0.338
Peak creatinine	158.4	171	155.3	
(μmol/L)	(115.0–255.6)	(122.1–266.5)	(113.0–253.0)	<0.001
Ultimate creatinine^a^	95	104.2	93.9	
(μmol/L)	(68.0–146.7)	(73.2–161.9)	(67.0–143.0)	<0.001
Oliguria/anuria^a^	833 (11.0)	170 (12.1)	663 (10.7)	0.125
AKI stage at the peak				
Stage 1	3483 (45.8)	631 (44.9)	2852 (46.0)	0.234
Stage 2	1950 (25.6)	385 (27.4)	1565 (25.2)	
Stage 3	2171 (28.6)	388 (27.6)	1783 (28.8)	
Critical condition^a^	3351 (44.1)	570 (40.6)	2781 (44.9)	0.004
RRT indication	896 (11.8)	179 (12.8)	717 (11.6)	0.214
AKI recognition type^a^				
Timely recognition	1604 (21.1)	327 (23.3)	1277 (20.6)	0.047
Delayed recognition	343 (4.5)	72 (5.1)	271 (4.4)	
Non recognition	5608 (73.8)	994 (70.8)	4614 (74.4)	
Short-term prognosis				
Renal recovery^a^				
Full recovery	2095 (33.2)	356 (32.0)	1739 (33.5)	0.022
Partial recovery	2071 (32.8)	338 (28.3)	1733 (31.7)	
Failed recovery	2139 (33.9)	419 (35.1)	1720 (31.4)	
Mortality^a^	927 (12.2)	208 (14.8)	719 (11.6)	0.001
Treatment withdrawal^a^	1617 (21.3)	277 (19.7)	1340 (21.6)	0.119
ICU stay (day)^a^	6 (3–14)	6 (3–15)	6 (3–14)	0.199
Length of stay (day)^a^	18 (10–29)	18 (11–32)	18 (10–29)	0.002
Hospitalization costs (RMB)^a^	31183.9	32771.6	30689	
	(14286.8–72719.9)	(15286.9–80113.8)	(13943.0–71906.2)	0.035

Data are expressed as mean (SD), *n* (%), or median (IQR), unless stated otherwise.

AKI, acute kidney injury; DM, diabetes mellitus; HA-AKI, hospital acquired acute kidney injury; CVD, cardiovascular disease; HBP, hypertension; CKD, chronic kidney disease; RRT, renal replace treatment; CEVD, cerebrovascular disease.

^a^ Data missing for CEVD in 1 patient (1 for patients without DM *vs*. 0 for patients with DM), ultimate creatinine in 981 patients (219 *vs*. 762), for oliguria/anuria in 57 patients (6 *vs*. 51), for AKI recognition type in 49 patients (11 *vs*. 38), for renal recovery in 1299 patients (291 *vs*. 1008), for mortality in 129 patients (21 *vs*. 108), for treatment withdrawal in 129 patients (21 *vs*. 108), for ICU stay in 5486 patients (4498 *vs*. 988), for length of stay in 5 patients (5 *vs*. 0), and for hospitalization costs in 1411 patients (1127 *vs*. 284).

### Short-term prognosis, length of stay, and costs for patients with DM and without DM

The results of clinical outcomes, length of stay, and cost for patients with and without DM are presented in Table 1. Approximately 30% for patients with AKI achieved full renal recovery before discharge in these two groups. Also, more cases of failed renal recovery were observed in the diabetic group (35.1% for patients with DM *vs*. 31.4% for patients without DM, *P* = 0.022). More in-hospital deaths occurred in DM patients than in those without DM (14.8% *vs*. 11.6%, *P* = 0.001). No apparent difference in treatment withdrawal was found between patients with and without DM in AKI (*P* = 0.119). Besides, treatment withdrawal was not associated with DM in the univariable regression model [odds ratio (OR) (95% confidence interval, 95% CI): 0.89 (0.77–1.03)]. Patients with DM experienced longer length of stay [median (IQR) 18 (11–32) *vs*. 18 (10–29), *P* <0.05)] and higher costs [(median (IQR) 32771.6 (15286.9–80113.8) *vs*. 30689.0 (13943.0–71906.2) Renminbi (RMB), *P* <0.05)] compared with patients without DM.

### Association between mortality, failed renal recovery, and DM history

The univariate and multivariate logistic regression models were used to estimate the correlations of all-cause in-hospital mortality and failed renal recovery with DM history. The univariate logistic regression analysis revealed that old age, CVD, HBP, CEVD, infection, more severe AKI stage at the peak, critical condition, antibiotics, and diuretics were significantly related to the in-hospital mortality (S1 Table). As shown in Table 2, patients with AKI and diabetes were more likely to encounter in-hospital mortality and failed renal recovery [odds ratio (OR) and 95% confidence interval (95% CI): 1.32 (1.12–1.56), 1.22 (1.07–1.39), *P* <0.01]. Female sex and location (southeast *vs*. north) were negative risk factors for all-cause in-hospital mortality in patients with AKI. The univariate analysis showed that old age, CVD, HBP, CKD, CEVD, infection, severe AKI stage at the peak, critical condition, antibiotics and diuretics were positively related to failed renal recovery (S2 Table). The inclusion criteria for covariates in this study were based on the study published by KJ Jager *et al*. [31] The covariates, including age, sex, region, CVD, HBP, CKD, CEVD, and diuretics were finally adjusted, according to their definition, in the multivariate models. As a result, DM history was not associated with all-cause in-hospital mortality [adjusted OR (95%CI): 1.16 (0.95–1.41), *P* = 0.15] and failed renal recovery in AKI patients after adjusted for the related covariates [adjusted OR (95%CI): 1.09 (0.95–1.26), *P* = 0.22)].

**Table 2:** Univariate and multivariate logistic regression analyses for the association of DM history with all-cause in-hospital mortality and failed renal recovery in patients with AKI.

	Expanded criteria			sKDIGO AKI criteria		
DM	Event number	OR (95% CI)	*P* value	Event number	OR (95% CI)	*P* value
All-cause in-hospital mortality^a^						
Model 1^c^	927	1.32 (1.12–1.56)	0.001	704	1.34 (1.01–1.64)	0.004
Model 2^d^	927	1.16 (0.95–1.41)	0.152	704	1.07 (0.84–1.36)	0.563
Failed renal recovery^b^						
Model 1^c^	2139	1.22 (1.07–1.39)	0.004	1346	1.30 (1.09–1.54)	0.003
Model 2^d^	2139	1.08 (0.94–1.25)	0.289	1346	1.08 (0.87–1.33)	0.478

^a^7475 and 3614 patients were included in the analysis after excluding 129 and 73 patients, whose information for all-cause in-hospital mortality was missing in expanded and KDIGO criteria models, respectively.

^b^ 6305 and 2831 patients were included in the analysis after excluding 1299 and 856 patients, whose information for renal recovery was missing in expanded and KDIGO criteria models, respectively.

^c^, Univariable logistic regression analysis.

^d^, Adjusted for age, gender, region, CVD, HBP, CKD, CEVD, infection, AKI stage in peak, critical condition, and diuretics.

AKI, acute kidney injury; DM, diabetes mellitus; CVD, cardiovascular disease; HBP, hypertension; CKD, chronic kidney disease; CEVD, cerebrovascular disease.

Further, a sensitivity analysis was conducted in patients diagnosed with AKI based on the KDIGO criteria in this survey. A total of 3687 patients were identified as having AKI using the KDIGO AKI criteria. As shown in Table 2, 19.1% and 36.5% patients succumbed to in-hospital death and failed renal recovery during hospitalization. The correlation of DM with in-hospital mortality [adjusted OR (95%CI): 1.07 (0.84–1.36)] and failed renal recovery [adjusted OR (95%CI): 1.08 (0.87–1.33)] were almost similar when AKI was diagnosed based on the 2012 KDIGO and expanded criteria in multivariate regression model.

### Association between length of stay, costs, and DM history

Table 3 depicts the association of unadjusted and multivariable-adjusted DM history with length of stay and costs in patients with AKI. Without adjustment, patients with DM required a longer length of stay and higher cost compared with those without DM. In multivariate regression model, preexisting DM showed positive associations with the length of stay and costs [*β* (95%CI): 0.07 (0.01–0.12), 0.11 (0.04–0.18), respectively; *P* <0.05].

**Table 3:** Univariate and multivariate linear regression analyses of Ln (length of stay) and Ln (hospital costs) in patients with AKI^A^.

DM	Regression coefficient	95% CI	P-value
Ln (length of stay)^a^			
Model 1^c^	0.11	0.06–0.16	<0.001
Model 2^d^	0.07	0.01–0.12	0.015
Ln (hospital costs)^b^			
Model 1^c^	0.10	0.03–0.17	0.008
Model 2^d^	0.11	0.04–0.18	0.003

^A^, Length of stay, hospital costs, and ultimate creatinine level were transformed by using logarithms to base e attributed to their skewness distribution.

^a^ 7599 patients were included in the analysis after excluding 5 patients, whose information for length of stay was missing.

^b^ 6193 patients were included in the analysis after excluding 1411 patients, whose information for hospital costs was missing.

^c^, Univariate linear regression analysis.

^d^, Adjusted for age, gender, region, CVD, HBP, CKD, CEVD, infection, AKI stage in peak, critical condition.

AKI, acute kidney injury; DM, diabetes mellitus; CVD, cardiovascular disease; HBP, hypertension; CKD, chronic kidney disease; CEVD, cerebrovascular disease.

## Discussion

The present study investigated the impact of diabetes on patients with AKI in China, including clinical outcomes and economic burden. Diabetes patients were older and had more comorbidities and higher creatinine levels (both peak and ultimate) in this study. Increased in-hospital mortality and less renal recovery were observed in patients with DM (14.8%, 60.3%) compared with those without diabetes (11.6%, 65.2%). A longer length of stay and higher hospitalization costs were found with increased complications of diabetes. DM was not associated with all-cause in-hospital mortality and failed renal recovery in patients with AKI after adjusting for relevant covariates. However, diabetes was positively related to the length of stay and costs in the hospital. These findings described an in-depth evaluation of the impact of diabetes on short-term prognosis, length of length of stay, and costs of patients with AKI in a developing country.

A recent study by Muroya *et al*. illustrated that the incidence and severity of AKI are increased in rats with type 2 diabetes [32]. In addition, a model of type 2 diabetes named Otsuka Long-Evans Tokushima Fatty rats suffered severe renal ischemia/reperfusion injury [33]. The present study found that the creatinine level significantly increased in patients with DM and AKI, indirectly resulting in poor renal outcomes. Also, *in vitro* and *in vivo* studies elaborated on the putative mechanisms of AKI onset in diabetes [34, 35]. Subsequently, a recent study found that the 1-year mortality of diabetes increased in patients with periprocedural AKI who underwent surgery [36]. However, DM was not independently associated with mortality in patients with AKI after adjusting for related covariates in this study. The possible reasons for this phenomenon were might be the “pre-conditioning” of the previous diabetic state through increased levels of hypoxia-inducible factor-1 alpha [37]. Another explanation for this result was the priming regeneration induced by hyperglycemia stimulation [34].

A significant finding of this study was an increased length of hospital stay and costs in patients with AKI and diabetes. Very few studies focused on the cost and length of stay of patients with diabetes and AKI. It was speculated that the abnormal vascular function and insulin resistance in patients with diabetes made them susceptible to severe comorbidities, such as disseminated intravascular coagulation and sepsis [38, 39], which increased the hospitalization costs and prolonged the length of stay of patients with DM and AKI. Fang *et al*. [40] revealed that the median length of stay and costs for patients with AKI were 21.2 days and 30764.3 RMB, respectively, which were consistent with the present findings. Moreover, the hospital costs for patients with DM exceeded those for patients without diabetes in present study. Also, DM remarkably increased the length of stay and expenditure of patients with AKI. This phenomenon could be partially attributable to the severe complications of diabetes during hospitalization, such as CVD, peripheral neuropathy, and CEVD [23, 41].

Nevertheless, the present study had several limitations. First, this retrospective study depended on repeated serum creatinine tests without urinary output records, leading to missed identification of AKI cases with inadequate serum creatinine tests or only with decreased urine output. Second, in clinical practice, severe patients visited the hospital and tended to receive more attention; therefore, mild cases were easily missed. This phenomenon might also cause the underestimation of the actual nonrecognition of AKI. Third, type 1 and type 2 DM could not be differentiated among patients with AKI in this study. Also, the effects of diabetes-related complications, glucose levels and diabetic control on AKI were missed. The poor control of diabetes easily caused serious microvascular and macrovascular complications, including diabetic nephropathy, diabetic retinopathy and diabetic neuropathy, leading to poor prognosis in patients with AKI. Fourth, this study included only 2 months of data. January and July were chosen to represent the winter and summer months in China due to some evidence to suggest there are seasonal effects of AKI [28] and it is unknown if this approach will over or underestimate associations between diabetes and AKI. Future studies should capture AKI across a full year. In addition, the covariates in multivariate analyses might be a part of the observations. Subsequently, the higher odds of failure to recover from AKI among patients with diabetes might be attributed to a high risk of mortality. Thus, a relatively comprehensive analysis of the impact of diabetes on the prognosis, hospitalization stay, and costs of patients with AKI was performed.

## Conclusions

In conclusions, this study showed that diabetes was significantly associated with an extended length of stay and increased medical costs in patients with AKI. Thus, the management of diabetes-related comorbidities in patients with AKI need an in-depth investigation.

## Supporting information

S1 Checklist STROBE statement. (DOCX)

S1 Table Univariate logistic regression analysis for the factors associated with all-cause in-hospital mortality in patients with AKI. (DOCX)

S2 Table Univariate logistic regression analysis of factors associated with failed renal recovery in patients with AKI. (DOCX)

S3 Table The diagnostic standard of the disease severity of AKI. (DOCX)
