# Supplementary material for: Impact of diabetes mellitus on short-term prognosis, length of stay, and costs in patients with acute kidney injury: A nationwide survey in China
Source: PLoS One. 2021 May 3;16(5):e0250934. doi: 10.1371/journal.pone.0250934 (PMC8092800; doi:10.1371/journal.pone.0250934)
Supplement: S1 Table — (DOCX) [file pone.0250934.s002.docx]

**S1 Table. Univariate logistic regression analysis for factors associated with all-cause in-hospital mortality in patients with AKI.**

| Factors | OR (95% CI) | *P*-value |
| --- | --- | --- |
| Age | 1.03 (1.02–1.03) | <0.001 |
| Sex |  |  |
| Male | Reference | – |
| Female | 0.77 (0.66–0.89) | <0.001 |
| Region |  |  |
| North | Reference | – |
| Southeast | 0.82 (0.70–0.98) | 0.024 |
| Northwest | 1.11 (0.88–1.40) | 0.394 |
| Southwest | 1.12 (0.91–1.38) | 0.277 |
| Any comorbidity | 1.21 (1.06–1.39) | 0.006 |
| CVD | 1.54 (1.34–1.77) | <0.001 |
| HBP | 1.34 (1.17–1.54) | <0.001 |
| CKD | 1.02 (0.87–1.20) | 0.811 |
| CEVD | 1.45 (1.22–1.71) | <0.001 |
| Infection | 1.62 (1.40–1.87) | <0.001 |
| AKI stage at the peak |  |  |
| Stage 1 | Reference | – |
| Stage 2 | 2.24 (1.87–2.69) | <0.001 |
| Stage 3 | 3.18 (2.68–3.76) | <0.001 |
| Critical condition | 7.13 (5.99–8.49) | <0.001 |
| Drugs |  |  |
| Antibiotics | 1.43 (1.25–1.64) | <0.001 |
| Diuretics | 2.09 (1.82–2.40) | <0.001 |
| NSAIDs | 1.20 (0.98–1.46) | 0.081 |
| Traditional Chinese medicine | 0.42 (0.19–0.89) | 0.024 |

AKI, Acute kidney injury; CEVD, cerebrovascular disease; CKD, chronic kidney disease; CVD, cardiovascular disease; HBP, hypertension; NSAID, non-steroidal anti-inflammatory drugs.
