# Supplementary material for: Impact of diabetes mellitus on short-term prognosis, length of stay, and costs in patients with acute kidney injury: A nationwide survey in China
Source: PLoS One. 2021 May 3;16(5):e0250934. doi: 10.1371/journal.pone.0250934 (PMC8092800; doi:10.1371/journal.pone.0250934)
Supplement: S2 Table — (DOCX) [file pone.0250934.s003.docx]

**S2 Table. Univariate logistic regression analysis for factors associated with failed renal recovery in patients with AKI.**

| Factors | OR (95% CI) | *P*-value |
| --- | --- | --- |
| Age | 1.00 (1.00–1.01) | <0.001 |
| Sex |  |  |
| Female | Reference | – |
| Male | 1.04 (0.94–1.16) | 0.445 |
| Region |  |  |
| North | Reference | – |
| Southeast | 0.93 (0.82–1.05) | 0.237 |
| Northwest | 0.99 (0.82–1.19) | 0.896 |
| Southwest | 0.85 (0.72–1.00) | 0.056 |
| Any comorbidity | 1.27 (1.14–1.40) | <0.001 |
| CVD | 1.23 (1.10–1.37) | <0.001 |
| HBP | 1.26 (1.13–1.40) | <0.001 |
| CKD | 1.38 (1.22–1.55) | <0.001 |
| CEVD | 1.03 (0.89–1.18) | 0.710 |
| Infection | 1.21 (1.09–1.35) | <0.001 |
| AKI stage at the peak |  |  |
| Stage 1 | Reference | – |
| Stage 2 | 0.49 (0.43–0.57) | <0.001 |
| Stage 3 | 0.69 (0.61–0.78) | <0.001 |
| Critical condition | 1.16 (1.05–1.29) | 0.006 |
| Drugs |  |  |
| Antibiotics | 1.13 (1.02–1.26) | 0.020 |
| Diuretics | 1.56 (1.41–1.74) | <0.001 |
| NSAIDs | 1.01 (0.86–1.19) | 0.916 |
| Traditional Chinese medicine | 0.84 (0.56–1.25) | 0.385 |

AKI, Acute kidney injury; CEVD, cerebrovascular disease; CKD, chronic kidney disease; CVD, cardiovascular disease; HBP, hypertension; NSAID, non-steroidal anti-inflammatory drugs.
