# Supplementary material for: Impact of diabetes mellitus on short-term prognosis, length of stay, and costs in patients with acute kidney injury: A nationwide survey in China
Source: PLoS One. 2021 May 3;16(5):e0250934. doi: 10.1371/journal.pone.0250934 (PMC8092800; doi:10.1371/journal.pone.0250934)
Supplement: S3 Table — (DOCX) [file pone.0250934.s004.docx]

S3 Table. The diagnostic standard of the disease severity of AKI.

| Stage | Serum creatinine | Urine volumn |
| --- | --- | --- |
| 1 | 1.5-1.9 times baseline OR ≥ 0.3 mg/dl (≥ 26.5µmol/L) | ＜0.5ml/kg/h for 6-12 hours |
| 2 | 2.0-2.9 times baseline | ＜0.5ml/kg/h for ≥ 12 hours |
| 3 | 3.0 times baseline OR Increase in serum creatinine to ≥4.0 mg/dl (≥353.6µmol/L) OR Initiation of renal replacement therapy. | ＜0.3 ml/kg/h for ≥ 24 hours OR Anuria for ≥ 12 hours |
